# Combined Effect of Resting Time and NaHCO_3_ on Solubility and Gel Properties of Low-Salt Chicken Myofibrillar Protein

**DOI:** 10.3390/foods14122121

**Published:** 2025-06-17

**Authors:** Wan-Li Cheng, Peng-Lei Yao, Xue-Hua Zhang, Yan-Yan Zhao, Sheng-Ming Zhao, Zhuang-Li Kang

**Affiliations:** 1 School of Food Science, Henan Institute of Science and Technology, Xinxiang 453003, China; c1751965688@163.com (W.-L.C.); zhaoyanayan@hist.edu.cn (Y.-Y.Z.); zhaoshengming2008@126.com (S.-M.Z.); 2 School of Tourism and Cuisine, Industrial Engineering Center for Huaiyang Cuisin of Jiangsu Province, Yangzhou University, Yangzhou 225127, China; 3 Key Laboratory of Chinese Cuisine Intangible Cultural Heritage Technology Inheritance, Ministry of Culture and Tourism, Yangzhou 225127, China

**Keywords:** whiteness, hardness, rheology property, solubility, aggregation

## Abstract

To investigate the interaction effects of resting times (0, 6, and 12 h) and NaHCO_3_ concentrations (0, 2, 4, and 6 g/kg) on chicken myofibrillar protein (CMP), this study analyzed the changes in solubility, active sulfhydryl groups, rheological behavior, fluorescence, and gel properties of CMP solutions (60 mg/mL). The results indicated that pH significantly increased with higher NaHCO_3_ concentrations and longer resting times. Consequently, solubility, active sulfhydryl groups, apparent viscosity, shear stress, G’ value at 80 °C, hardness, springiness, and cohesiveness all significantly increased, while particle size, turbidity, and whiteness significantly decreased. However, these trends were not observed in samples treated with an amount of 6 g/kg NaHCO_3_ and/or a resting time of 12 h. The findings suggest that treatment with 4 g/kg NaHCO_3_ and a resting time of 6 h effectively reduced protein aggregation and enhanced solubility. Conversely, excessive NaHCO_3_ or prolonged resting times resulted in decreased protein solubility and deteriorated textural properties.

## 1. Introduction

The World Health Organization recommends that adults consume no more than 5 g of salt daily. Excessive sodium chloride intake is linked to increased risks of hypertension, cardiovascular disease, renal disease, and gastric cancer [1], driving growing public interest in low-salt diets. Meat products account for approximately 25% of daily salt intake, making them the second largest source [2]. Sodium bicarbonate (NaHCO_3_) serves as an alternative to sodium chloride due to its moderate alkalinity, water solubility, buffering capacity, and suitability for meat processing [3]. Studies show that adding 0.1–0.8 g/100 g NaHCO_3_ can improve tenderness; reduce cooking loss; and enhance taste, color, and texture in pork and chicken breast by raising pH and ion strength [3,4]. Furthermore, adding 0.1–0.6 g/100 g NaHCO_3_ also modifies myofibrillar protein structures, increasing solubility and processing quality [5,6].

Myofibrillar protein is the predominant component of meat, constituting approximately 55% of the total protein content [7]. The interactions between myofibrillar proteins, known as aggregation and cross-linked, are influenced by factors such as temperature, pH, and ionic strength [8,9]. When the pH of the protein approaches its isoelectric point (5.40–5.50), the repulsive forces between myofibrillar proteins weaken, leading to an increased tendency to aggregate and a subsequent decrease in solubility [10]. Added 0.1~0.6 g/100 g NaHCO_3_ has been shown to modify the isoelectric point of proteins by increasing hydroxide ion concentration, enhancing myofibril hydration, and inducing its swelling [11]. Zhu et al. [12] demonstrated that adding 0.5% NaHCO_3_ significantly raised the pH of chicken batter, increased the solubility of myofibrillar proteins, altered their secondary and tertiary structures, and improved low-sodium susceptible gelatin quality. Similarly, some researchers [13,14] reported that the increase in concentrations of NaHCO_3_ from 0 g/100 g to 0.6 g/100 g weakened actin–myosin interactions and significantly deactivated ATPase activity while increasing protein solubility, regardless of sodium chloride presence or absence.

The common process in factory production is to marinate the processed meat batter at 4 °C for 0 to 12 h. The main reason might be that the extraction rate of salt-soluble proteins and their movement towards the meat surface stabilize within 10 to 18 h, ensuring that the salt-soluble proteins are fully extracted and the entire emulsification system is relatively stable. However, as the resting time increases, the emulsification system of the meat batter may become damaged, with small bubbles aggregating to form larger ones. The partial separation of oil and water occurs in the emulsification system, and the partial contraction of the spatial structure of salt-soluble proteins results in the poor water retention of the product [8,15,16]. Nonetheless, there are relatively few studies on the mechanism by which resting time affects the quality of minced meat. Inspired by these findings, this study systematically investigated the effects of NaHCO_3_ (0, 2, 4, and 6 g/kg) on the solubility, protein structure, aggregation, and gel properties of CMP with different resting times (0, 6, and 12 h) in a low-salt environment. It aimed to analyze in depth the mechanism of NaHCO_3_ which affects the protein structure and conformation.

## 2. Materials and Methods

### 2.1. Materials and Ingredients

After slaughtering and cooling the broilers (2500 ± 200 g, AA broiler, 42 d) for 5 h, the chicken breasts were frozen them at −38 °C to a center temperature of −18 °C. A total of 30 kg frozen chicken breast meat was purchased 3 times on 3 days from Zhengda Food Enterprise Co., Ltd. (Qingdao, China), then stored at −20 °C and used within 30 d. NaHCO_3_ and the other chemical reagents were all of analytical purity.

### 2.2. Extraction of Chicken Myofibrillar Protein (CMP)

The extraction of CMP was performed according to our previous method [3]. In short, the crude myofibrillar protein was obtained after the ground chicken was washed three times using four volumes of phosphate-buffered saline (100 mmol/L KCl, 20 mmol/L Na_2_HPO_4_/NaH_2_PO_4_, 2 mmol/L MgCl_2_, 1 mmol/L EGTA, 1 mmol/L NaN_3_, pH 7.0). The obtained residue was washed twice with four volumes of low-salt solution (0.1 mol/L NaCl), filtered with four layers of gauze (pH 6.0), and the resulting residue was pure CMP.

### 2.3. The Preparation of CMP Solutions and Gels

According to our previous paper [15], the CMP was diluted to 60 mg/mL with 1 mmol/L NaCl and mixed with 0 g/kg, 2 g/kg, 4 g/kg, and 6 g/kg NaHCO_3_, respectively. The treatment groups with different NaHCO_3_ addition amounts and resting times (0, 6, and 12 h) were named as T1 (0 h, 0 g/kg NaHCO_3_), T2 (0 h, 2 g/kg NaHCO_3_), T3 (0 h, 4 g/kg NaHCO_3_), T4 (0 h, 6 g/kg NaHCO_3_), T5 (6 h, 0 g/kg NaHCO_3_), T6 (6 h, 2 g/kg NaHCO_3_), T7 (6 h, 4 g/kg NaHCO_3_), T8 (6 h, 6 g/kg NaHCO_3_), T9 (12 h, 0 g/kg NaHCO_3_), T10 (12 h, 2 g/kg NaHCO_3_), T11 (12 h, 4 g/kg NaHCO_3_), and T12 (12 h, 6 g/kg NaHCO_3_), respectively. After standing for 0 h, 6 h, and 12 h at 4 °C, two sampling spoons were used to remove 8 g of chicken myofibrin solution and place them in a 10 mL beaker. After heating them in an 85 °C water bath for 30 min, they were removed and cooled to room temperature, then put in the 4 °C refrigerator overnight.

### 2.4. Determination of pH

The solution of each group was diluted to 5 mg/mL; then 20 mL was homogenized at the corresponding treatment temperature of the proteins for 10 s with a 1500 rpm homogenizer (Xinzhi Biotechnology Ltd.; Ningbo, China) and measured with a portable pH meter (digital, Hanna, Milan, Italy).

### 2.5. Determination of Particle Size

The CMP solution was diluted to 0.1 mg/mL, and the particle size was analyzed by a Zetasizer v7.11 laser nanoparticle size analyzer (Malvern Company, Malvern, UK).

### 2.6. Determination of Turbidity

The concentration of CMP solution was adjusted to 1 mg/mL, and then the absorbance of each group was measured at 600 nm by ultraviolet spectrophotometer.

### 2.7. Determination of Solubility

Determination of total solubility: 1 mL of CMP solution (5 mg/mL) and 4 mL of biuret solution reacted for 30 min in the dark, and the absorbance was measured at 540 nm with a 722N visible spectrophotometer(Shanghai Huyueming Scientific Instruments Co., Ltd., Shanghai, China).

Solubility determination of the supernatant: 5 mL of CMP solution was taken and centrifuged at 10,000× *g* for 30 min to obtain the supernatant. A total of 1 mL of supernatant and 4 mL of urea and protect from light for 30 min. The absorbance was measured at 540 nm using a mixture containing 1 mL of distilled water and 4 mL of urea as a control. The protein concentrations were calculated using bovine serum standard curves and named S1 (total protein solubility) and S2 (supernatant solubility), respectively. The solubility of myofibrillar protein in each treatment group was calculated according to Equation (1).

(1) $$Myofibrillar\;protein\;solubility\;(\%)=\frac{S2}{S1}\;\times \;100$$

### 2.8. Determination of Active Sulfhydryl Groups

According to the method of Kang et al. [15], the active sulfhydryl content in each treatment group was calculated.

### 2.9. Determination of Fluorescence Properties

The measurement of fluorescence properties, using the method of Kang et al. [5], was carried out utilizing a G9800A fluorescence spectrophotometer (Agilent Company, Lexington, MA, USA).

### 2.10. Determination of Apparent Viscosity and Shear Stress

The 60 mg/mL CMP treatment solution was evenly spread on the plate with a thickness of 1 mm and was measured using a P35 TiL probe (Thermo Scientific Ltd.; Dreieich, Germany). The shear rate was set to 10 s^−1^~1000 s^−1^, the temperature was 25 °C, the shear time was 330 s, the parameter was set to 100 points, and the fixed frequency was 0.1 Hz.

### 2.11. Determination of Rheological Property

Utilizing the method of Zhu et al. [12], a Hake MarsIII rotational rheometer (Thermo Scientific Ltd., Germany) equipped with a P35 TiL probe was used to perform a variable temperature scan of the CMP treatment solution at 60 mg/mL. The gap between the plate and the probe was set to 1 mm, the CMP solution was slowly flattened, the excess sample was wiped off, the outermost layer was coated with a layer of silicone oil to seal, and then it was kept warm at 20 °C for 5 min. The heating rate was set to 2 °C/min, the frequency was 0.1 Hz, the temperature range was 20–80 °C, and the changes in storage modulus (G′) was recorded.

### 2.12. Determination of Whiteness

The whiteness of the CMP gel was determined by a CR-400 colorimeter (Konica Minolta Ltd.; Tokyo, Japan). The color difference meter was corrected by a standard whiteboard with L^*^ = 96.51, a^*^ = −0.87, and b^*^ = −1.48. The gel was into 0.3 cm thin slices and the measurement of the color of the cut surface was completed within 3 min. The whiteness of CMP gel was calculated according to the following Equation (2).

(2) $$Whiteness=100-\sqrt{{(100\;-\;L^{*})}^{2}+({a^{*})}^{2}+({b^{*})}^{2}}$$

### 2.13. Determination of Texture

After leaving them overnight, CMP gels were measured with a texture analyzer equipped with a P36/R probe (TA-XT plus, Stable Micro System Ltd., Surry, UK) [5]. The pre-test speed was 1.0 mm/s, the test speed was 0.5 mm/s, the post-test speed was 10 mm/s, and the compression ratio was 50%. Finally, changes in the hardness, springiness, and cohesiveness of the samples were recorded.

### 2.14. Statistical Analysis

Three independent repetitions (*n* = 3) were performed in accordance with different source materials. Analysis was performed using SPSS v.26.0 statistical software (SPSS Inc., Chicago, IL, USA) and origin 2022 mapping software, all results are expressed as mean ± standard deviation. The data were analyzed by one-way analysis of variance (ANOVA) and the general linear model (GLM) procedure using the SPSS V.18.0, with the treatments considered the fixed effects and the replicates the random effects. The significant differences between means *(p* < 0.05) were determined by Duncan’s Multiple Range Test.

## 3. Results and Discussion

### 3.1. pH

**Figure 1:**
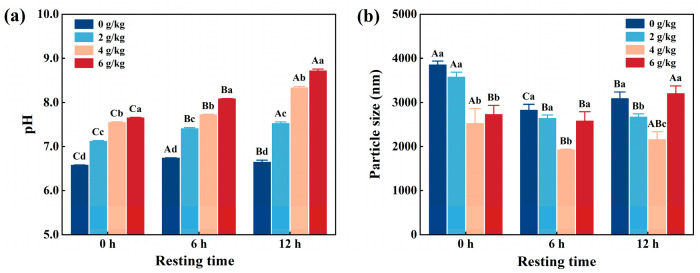
Effects of NaHCO_3_ addition and resting time on pH (**a**) and particle size (**b**) of low-salt chicken myofibrillar protein. Each value represents the mean ± SE, *n* = 3. ^A–C,a–d^ Different parameter superscripts indicate significant differences (*p* < 0.05).

Figure 1a shows that the pH of CMP significantly (*p* < 0.05) increased from 6.61 to 8.36 with increasing NaHCO_3_ addition during the same resting time. Similarly, the pH of CMP significantly (*p* < 0.05) increased with increasing resting time when NaHCO_3_ was added at a constant level. It is well known that NaHCO_3_ decompose easily and produces acid ions when it comes into contact with water, leading to an increase in the pH of CMP solutions [16]. Similarly, Zou et al. [3] reported that added NaHCO_3_ from 0 to 0.6% significantly increased the pH of the meat batter. Kang et al. [17] reported that the pH increased with increasing NaHCO_3_.

### 3.2. Particle Size

Particle size is an essential indicator for analyzing the myofibrillar protein [18]. As shown in Figure 1b, the particle size of CMP decreased significantly (*p* < 0.05) from 3892 nm to 1979 nm with increasing NaHCO_3_ addition for the same resting duration. Similarly, the particle sizes also decreased significantly (*p* < 0.05) with increasing resting time under the same NaHCO_3_ additions, except for the sample of resting for 12 h. The smallest value was observed when 4 g/kg NaHCO_3_ was added and the mixture rested for 6 h. This phenomenon can be attributed to the gradual increase in the pH of the CMP as resting time increased. The reason for this is that an increase in pH weakens the myofibrillar protein due to the blocking of electrostatic interactions between proteins, leading to a reduction in particle size [12]. By contrast, due to the change in structure of the myofibrillar proteins transformed by the high pH, the particle size increased when NaHCO_3_ was added to 6 g/kg of chicken and the resting period was 12 h. Similarly, Zou et al. [2] found that the particle size of myofibrillar protein from PSE pork gradually decreased with increasing NaHCO_3_ addition. On the other hand, Lametsch et al. [19] showed that pork myofibrillar protein rested for 8 d had a smaller particle size than the sample which rested for 1 d. Thus, when the resting time was continuously extended, there was a possibility that the proteins precipitated and coagulated, which might have an undue effect on the particle size.

### 3.3. Turbidity

**Figure 2:**
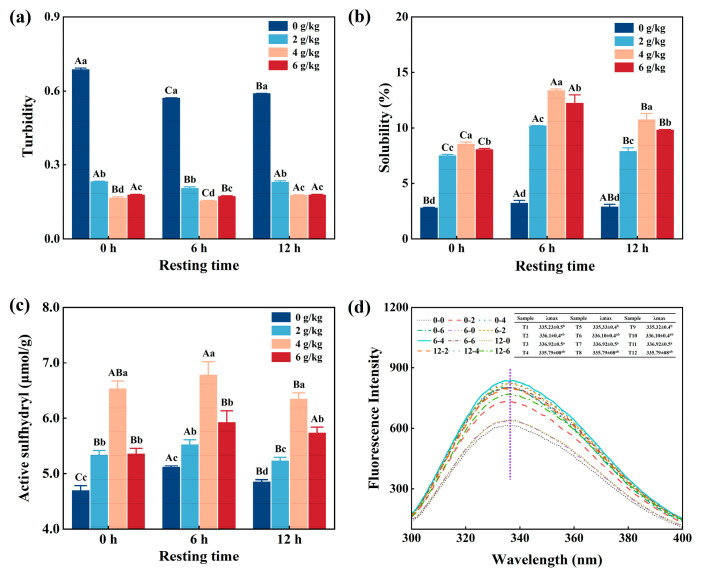
Effect of NaHCO_3_ addition and resting time on turbidity (**a**), solubility (**b**), active sulfhydryl groups (**c**), and the endogenous fluorescence spectrum (**d**) of low-salt chicken myofibrillar protein. Each value represents the mean ± SE, *n* = 3. ^A–C,a–d^ Different parameter superscripts indicate significant differences (*p* < 0.05).

As shown in Figure 2a, the turbidity of CMP decreased gradually (*p* < 0.05) from 0.72 to 0.24 with increasing NaHCO_3_ addition under the same resting times. Similarly, the turbidity decreased significantly (*p* < 0.05) with increasing resting time under the same NaHCO_3_ addition rates. When the NaHCO_3_ addition was 4 g/kg and the resting time was 6 h, the turbidity reached its lowest rate, at 0.24. It is well known that due to the weakly base nature of NaHCO_3_, it can be dissolved in a solution system and decomposed to produce OH^-^ and HCO_3_^−^. This process increased pH, thereby weakening the intermolecular interactions, which reduced the turbidity of the CMP solution [17]. It was not ideal that if the resting time was too long or the NaHCO_3_ content was too high, the CMP was over-expanded and/or the pH rose to a certain extent, which may lead to changes in the intermolecular interaction of myofibrillar protein, which increased the turbidity of the protein [20].

### 3.4. Solubility

Figure 2b shows that the solubility increased significantly (*p* < 0.05) with increasing NaHCO_3_ under the same resting times; it also increased significantly with increasing resting times under the same NaHCO_3_ addition rate, and the highest value was produced when the NaHCO_3_ addition was 4 g/kg and the resting time was 6 h. The reason for this is that the addition of NaHCO_3_ affected the electrostatic interaction of the protein, thereby changing the conformation of the protein and increasing the solubility [21]. However, too high a pH and long resting times can structurally disrupt the myofibrillar protein [22], and an irreversible denaturation of the protein and a subsequent reduction in solubility can occur when the NaHCO_3_ addition is 6 g/kg and the resting time is 12 h.

### 3.5. Active Sulfhydryl Groups

Sulfhydryl groups are the most active functional groups and contribute to the maintenance of the three-dimensional structure and improve their thermal stability [23]. Figure 2c shows that the active sulfhydryl groups of CMP increased significantly (*p* < 0.05) with increasing NaHCO_3_ addition under the same resting times and also increased significantly (*p* < 0.05) with increasing resting times under the same NaHCO_3_ addition rate; it reached maximum when the NaHCO_3_ addition was 4 g/kg and the resting time was 6 h. This is because NaHCO_3_ can reduce the forces between proteins and accelerate the hydrolysis of myofibrillar proteins, leading to the exposure of more of the sulfhydryl groups within the proteins to the surface of the molecule. In addition, there is the potential for the myofibrillar protein environment to be altered when the resting time is prolonged, resulting in the disruption of the disulfide bonds. The partial embedding of exposed active sulfhydryl groups when treated with an excessively high concentration of NaHCO_3_ or/and an extended period of rest results in a reduction in the content of the active sulfhydryl groups [24,25].

### 3.6. Fluorescence Properties

Fluorescence properties can be used to demonstrate that the myofibrillar proteins are mainly derived from tyrosine, tryptophan, and phenylalanine residues, the most prevalent of these are tryptophan residues [26]. As shown in Figure 2d, the maximum fluorescence nature wavelength (λmax) of tryptophan was approximately 336 nm. In addition, the fluorescent properties of CMP had different responses to the different NaHCO_3_ addition and resting times. The sample that had a 4 g/kg NaHCO_3_ addition and a resting time of 6 h had the largest fluorescence intensity, because NaHCO_3_ resulted in the tertiary structure of the CMP opening and exposing the aromatic amino acids inside it to the polar environment [14,17]. However, when the amount of NaHCO_3_ was over 4 g/kg, the environment of the CMP was more alkaline, which caused the myofibrillar protein to over-expand and re-crosslink, resulting in a decrease in fluorescence intensity. Moreover, when resting time was too long, a more thorough reaction occurred between NaHCO_3_ and/or CMP, which affected the fluorescence intensity and wavelength of the protein through changes in the protein environment and tryptophan exposure [27].

### 3.7. Apparent Viscosity and Shear Stress

**Figure 3:**
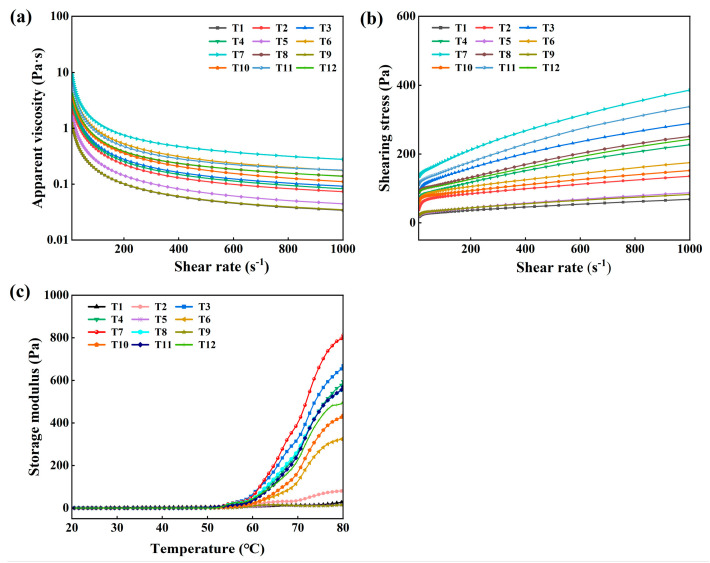
Effect of NaHCO_3_ addition and resting time on the apparent viscosity (**a**), shear stress (**b**), and rheological properties (**c**) of low-salt chicken myofibrillar protein.

As shown in Figure 3a,b, the apparent viscosity tended to decrease with increasing shear rate. This is because the internal structure of myofibrillar protein molecules was gradually destroyed or reorganized as the shear rate increased. This resulted in a decrease in the intermolecular interaction force, an increase in the distance between the molecules, and an increase in the molecules’ tendency to flow, which consequently reduced the apparent viscosity of myofibrillar proteins with an increase in the shear rate [28,29]. Under the same shear rate condition, the sample with a 4 g/kg NaHCO_3_ addition and a resting time of 6 h had the highest apparent viscosity and shear stress, which may be attributed to the fact that NaHCO_3_ increased pH and active sulfhydryl groups, resulting in higher viscosities. Previous studies showed that the disruption of disulphide and hydrogen bonds between myofibrillar protein molecules is the primary cause of the observed reduction in apparent viscosity [30,31]. However, excessive NaHCO_3_ led to an increase in pH of CMP, which was able to weaken the protein interactions.

### 3.8. Rheology

Rheological property is an important indicator of protein assays, where the G’ represents the energy stored by the elastic fraction of the protein [32]. As shown in Figure 3c, the denaturation of myofibrillar protein is divided into three stages. In the first stage, the G’ showed a slow upward trend under 52 °C and formed the “gel weakening phase” caused by the degeneration of the myosin head as temperature increased [33]. In the second stage, the G’ showed a rapid upward trend over 52 °C caused by the aggregation and denaturation of the myosin tail [34]. Due to the CMP myofibrillar proteins having weaker intermolecular interactions, larger intermolecular gaps, and looser molecular structures at low-salt concentrations, no significant peaks were formed. In the third stage, the aggregation of the proteins formed a three-dimensional network at 80 °C [35]. At 80 °C, the sample with a rate of 4 g/kg NaHCO_3_ and a resting time of 6 h has the highest G’, because it has the highest solubility. Similarly, Zhu et al. [12] reported that the two stages of the protein denaturation of chicken batter with NaHCO_3_ occurred.

### 3.9. Whiteness

**Figure 4:**
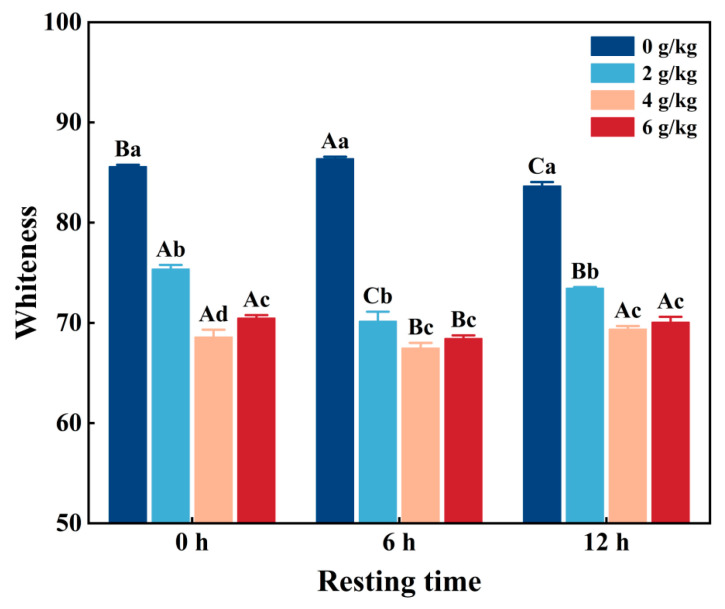
Changes in the whiteness of low-salt chicken myofibrillar protein gels under different NaHCO_3_ addition rates and resting times. Each value represents the mean ± SE, *n* = 3. ^A–C,a–d^ Different parameter superscripts indicate significant differences (*p* < 0.05).

The whiteness value plays a key role in the evaluation of myofibrillar protein gels and helps to ensure their optimal quality. As shown in Figure 4, the whiteness value of CMP decreased significantly (*p* < 0.05) with increasing NaHCO_3_ addition and/or resting time. Due to NaHCO_3_ being able to change the interactions between myofibrillar protein and its gel through increases in pH, thereby reducing the whiteness value [36], the sample with the addition of 4 g/kg NaHCO_3_ and resting time of 6 h has the lowest whiteness value. In addition, it was shown that the whiteness of the gel depends on the degree of denaturation of the myofibrillar protein and its water-holding capacity, because the deterioration of the reticular structure of the protein gel led to a decrease in its ability to reflect light and a decrease in whiteness [37]. However, when the NaHCO_3_ addition was over 4 g/kg and the resting time was over 6 h, protein aggregation occurred (Figure 2a), increasing the whiteness value of the gel. A previous study found that NaHCO_3_ darkens the color of gel during heating [38]. Kang et al. [5] reported that increased NaHCO_3_ addition significantly decreased the whiteness of reduced-salt pork myofibrillar protein gel.

### 3.10. Texture Properties

As shown in Table 1, the hardness, springiness, and cohesiveness of CMP gel increased significantly (*p* < 0.05) with increasing NaHCO_3_ addition or/and resting time, except in the sample with 6 g/kg NaHCO_3_ and resting time of 12 h. The sample with 4 g/kg NaHCO_3_ and resting time of 6 h had the highest hardness, springiness, and cohesiveness rates. The result indicated that increased NaHCO_3_ addition and/or resting time can elevate pH and solubility, forming more active sulfhydryl groups, which promotes protein cross-linking during heating. Wu et al. [39] demonstrated that through incorporating higher amounts of NaHCO_3_, the hardness, elasticity, and cohesion of myofibrillar protein gels were significantly enhanced. However, excess NaHCO_3_ addition and resting time caused a decrease in protein solubility, which reduced the textural properties. Previous research found that an appropriate addition of NaHCO_3_ increased the solubility of myofibrillar protein, thereby reducing the adverse effects of the thermal denaturation of its protein [40].

## 4. Conclusions

Based on the aforementioned studies, it is evident that the addition of NaHCO_3_ and the resting time significantly influence the structure and aggregation of CMP. As the resting time and NaHCO_3_ concentration increased, a marked rise in pH was observed, causing the CMP to progressively deviate from its isoelectric point. This deviation resulted in enhanced solubility and active sulfhydryl groups, as well as reduced particle size and turbidity. The maximum values for solubility, active sulfhydryl groups, apparent viscosity, shear stress, G’ at 80 °C, and texture properties were achieved with the addition of 4 g/kg NaHCO_3_ and a resting time of 6 h. Conversely, the minimum values for particle size, turbidity, and whiteness were also recorded under these conditions. However, when the NaHCO_3_ concentration was increased to 6 g/kg and/or the resting time extended to 12 h, the solubility, active sulfhydryl groups, rheological properties, and texture characteristics decreased. In conclusion, the combination of 4 g/kg NaHCO_3_ and a 6 h resting time demonstrated the most pronounced effect on the solubility and gel properties of CMP.

## Figures and Tables

**Table 1:** Effect of NaHCO_3_ addition and resting time on the texture of low-salt chicken myofibrillar protein gels.

Samples	Hardness (g)	Springiness	Cohesiveness
T1	22.08 ± 0.79 ^e^	0.79 ± 0.06 ^d^	0.42 ± 0.02 ^e^
T2	94.98 ± 3.81 ^c^	0.92 ± 0.01 ^c^	0.52 ± 0.06 ^d^
T3	160.83 ± 20.55 ^a^	0.96 ± 0.00 ^b^	0.75 ± 0.02 ^a^
T4	109.67 ± 30.20 ^bc^	0.94 ± 0.01 ^b^	0.75 ± 0.02 ^a^
T5	49.69 ± 3.35 ^d^	0.73 ± 0.03 ^d^	0.44 ± 0.02 ^e^
T6	134.18 ± 6.06 ^b^	0.95 ± 0.02 ^b^	0.73 ± 0.02 ^b^
T7	187.14 ± 5.69 ^a^	0.99 ± 0.01 ^a^	0.77 ± 0.01 ^a^
T8	121.76 ± 7.88 ^b^	0.96 ± 0.01 ^b^	0.74 ± 0.04 ^ab^
T9	20.79 ± 2.39 ^e^	0.66 ± 0.01 ^e^	0.48 ± 0.01 ^d^
T10	108.10 ± 18.35 ^c^	0.94 ± 0.03 ^b^	0.67 ± 0.04 ^c^
T11	127.44 ± 24.25 ^bc^	0.95 ± 0.01 ^b^	0.77 ± 0.01 ^a^
T12	111.74 ± 17.80 ^b^	0.95 ± 0.01 ^b^	0.72 ± 0.04 ^ab^

Each value represents the mean ± SE, *n* = 3. ^a–e^ Different parameter superscripts indicate significant differences (*p* < 0.05).

## Data Availability

The original contributions presented in the study are included in the article, further inquiries can be directed to the corresponding author.
